# Compassion-focused therapy with autistic adults

**DOI:** 10.3389/fpsyg.2023.1267968

**Published:** 2023-10-26

**Authors:** David Mason, James Acland, Eloise Stark, Francesca Happé, Debbie Spain

**Affiliations:** ^1^Social, Genetic and Developmental Psychiatry Centre, Institute of Psychiatry, Psychology & Neuroscience, King's College London, London, United Kingdom; ^2^South London and Maudsley NHS Foundation Trust, London, United Kingdom; ^3^Oxford Institute of Clinical Psychology Training and Research, Medical Sciences Division, University of Oxford, Oxford, United Kingdom; ^4^Centre for Eudaimonia and Human Flourishing, Stoke House, Linacre College, University of Oxford, Oxford, United Kingdom

**Keywords:** autism, compassion-focused therapy, shame, threat system, drive system, soothe system

## Abstract

Some autistic adults experience repeated adverse events, including rejection, victimization and stigmatization. They also describe others being critical and negatively judging them, such as for how they socially interact or for expressing passion for particular interests. The impact of these adverse events can be substantial, including increasing vulnerability for poorer mental health, and contributing to development of negative self beliefs (such as “I am different” or “I do not fit in”) and shame-based difficulties. Not all evidence-based psychological therapies are well-received by autistic people, or effective. Given high rates of self-harm and suicidality, finding acceptable and effective therapies for autistic adults is paramount. Here, writing as autistic and non-autistic clinicians and researchers, we outline the theoretical principles of compassion-focused theory and therapy (CFT). We propose that: (1) compassion-focused theory can provide a useful framework for conceptualizing shame-based difficulties some autistic adults experience; (2) CFT can be appropriate for addressing these; and (3) there is an impetus for practitioners to adopt compassion-focused approaches when supporting autistic adults.

*“At primary school other kids ignored me. At secondary school, I was bullied. The teachers told me to try harder to fit in. I didn’t know how.”*

*“I go to the bathroom to stim. My mum always says people will think it’s weird, that I’m weird. It calms me down, but she thinks I should stop doing it.”*

*“I force myself to go to the pub with friends. They say I’ll enjoy it. I don’t. I need a few drinks beforehand, put on a mask, pretend to be normal. The noises, smells, people, it’s overwhelming. It takes days to recover.”*

## Mental health of autistic adults

Many autistic adults experience poor mental health (Lai et al., 2019). There is burgeoning interest in transdiagnostic characteristics potentially contributing to this, including emotion regulation (Cai et al., 2018), intolerance of uncertainty (Jenkinson et al., 2020), and sensory sensitivity (Glod et al., 2015). Broader characteristics associated with mental health in neurotypical adults are less commonly researched, with shame being one such example (Gilbert, 2014).

Shame is insidious. In neurotypical adults, shame-based difficulties exacerbate poor mental health, can result in risky coping strategies including self-harm (Kim et al., 2011; Cândea and Szentagotai-Tătar, 2018; Sheehy et al., 2019), and impact effectiveness of psychological therapies (Gilbert, 2009a). Some autistic adults can be self-critical and experience shame, in part due to invalidating and traumatic past and ongoing events (Acland and Spain, 2022). In clinical practice, we find that some autistic adults benefit from compassion-focused therapy (CFT); an evidence-based approach that addresses the impact of adverse experiences and shame-based difficulties (Gilbert, 2014; Craig et al., 2020).

Here, we provide an overview of CFT. We outline why this may hold relevance for understanding and addressing shame-based difficulties some autistic adults experience, and propose that practitioners adopt a compassion-focused approach when supporting autistic adults.

## Principles underpinning compassion-focused therapy

CFT is a biopsychosocial approach, drawing on evolutionary, neuropsychological, cognitive-behavioral, social and attachment theories (see Gilbert, 2014, 2017). CFT aims to support people to develop “sensitivity to suffering in self and others, [and] a commitment to try to alleviate and prevent it” (Gilbert, 2014, pp.19).

The approach is underpinned by key tenets. The first is that human brains have evolved over millions of years, comprising the ‘old’ mammalian part primed to focus on basic needs and the ‘new’ part focused on higher level skills in the pre-frontal cortex, including planning, reflecting and imagining (Gilbert, 2017). These two parts are complementary, helping us to function in the modern world. For example, we can react rapidly in some contexts and be reflective in others. However, our brains can be a bit ‘tricky’, through no fault of our own. Sometimes, the ‘old’ and ‘new’ parts get caught up in loops, resulting in us anticipating threats that contribute to us becoming stuck (Gilbert, 2014). Within the highly detailed mind of an autistic adult, this loop can be more pronounced, resulting in elevated anxiety (South and Rodgers, 2017).

Second, it is proposed that humans have an emotion (affect) regulation system, comprising threat, drive and soothe systems (Gilbert, 2009a). The threat system is our internal warning system. When faced with threat or danger, negative emotions (primarily anxiety, anger, disgust) come quickly to the fore, to ensure we respond in safety-maximizing ways. The drive system focuses on doing and achieving. It propels us toward potentially useful goals, rewards and resources, and links with positive emotions (e.g., excitement, pleasure). The soothe system is the antithesis of threat and drive, helping to downregulate these. It becomes activated when we feel safe (or we are learning to feel safe), can slow down and develop affiliative relationships, and is associated with contentment and calmness (known as ‘rest and digest’; Gilbert, 2017).

When our emotion regulation system is balanced, different systems come online flexibly and adaptively, depending on the situation. Yet, for some people, these systems are over-active, under-developed or imbalanced. For example, victimization or ostracism at school, a commonly reported experience of autistic adults, can result in a sensitized threat system and hypervigilance for imminent (social) danger. Similarly, abuse or neglect from caregivers can result in an under-developed soothe system and over-active drive system, with the person striving to perform well in order to demonstrate their capabilities to others. Adverse early experiences and limited opportunities to feel safe and connected to others can mean the soothe system is disrupted. Together, an unbalanced emotion regulation system can result in distress and disconnection.

Third, affiliative social relationships – characterized by caring, sharing, closeness and safeness – are considered critically important for wellbeing (Gilbert, 2015). Historically, humans started developing cooperative rather than competitive relationships in hunter–gatherer societies; working together to improve chances for survival (e.g., finding food, shelter). Cohesive and caring relationships are thus deemed beneficial proactively (e.g., giving us a chance to learn from others, gain confidence, be playful), and reactively (e.g., managing difficulties or danger together, downregulating the threat system to facilitate wise responses to threats) (Gilbert, 2022a). They provide a way for people to feel part of, rather than separate from, others. This is important because social disconnection, isolation, poor attachment and perceptions of inferiority or difference, are risk factors for poorer health (McEwan et al., 2012).

Fourth, the brain/mind is full of competing and conflicting motives, needs and emotions (Gilbert, 2015). However, positive and challenging life experiences can influence how much our motives, needs and emotions compete and conflict, what we think about our needs and how we respond to these (Irons, 2019). For some people, sensitivity to their emotions, and the wisdom to respond to these safely, conflicts with how others have shown emotion to them (e.g., if others have invalidated emotions or shown inconsistent responses). Allowing individuals to rest and use compassionate qualities is especially important.

Finally, a universal, cross-cultural human concern is how to cope with inevitable life challenges (Pigliucci, 2017). Instinctively, we try to protect ourselves and others from these. Sometimes our strategies provide a quick fix, rather than a longer-term solution (e.g., as in the case of avoidance or disconnection). These are understandable strategies. However, they do not facilitate emotion processing, and can unintentionally encourage over-active threat and drive systems, and increase vulnerability for poorer mental health (Gilbert, 2014; Irons, 2019). As an alternative, qualities of wisdom and courage can enhance our strength to manage these challenges (Gilbert, 2009a). Supporting autistic adults to work toward seeing themselves as equal, included and different to those around them, can lead to compassionate behaviors that reduce both current feelings of shame, and vulnerability to future potential shame-based or invalidating experiences.

## Compassion-focused therapy in practice

In practice, CFT involves understanding more about different factors potentially implicated in the development of an unbalanced (disrupted) emotion regulation system (e.g., traumatic experiences), and how these experiences may have contributed to shame-based difficulties. This relates to a further CFT tenet; many factors were beyond our control, as these stem from earlier (adverse) life events or have an evolutionary basis. In this way, CFT is a ‘deshaming’ approach; highlighting that the way a person’s brain and emotion regulation system work is not their fault (Gilbert, 2014).

**Figure 1:**
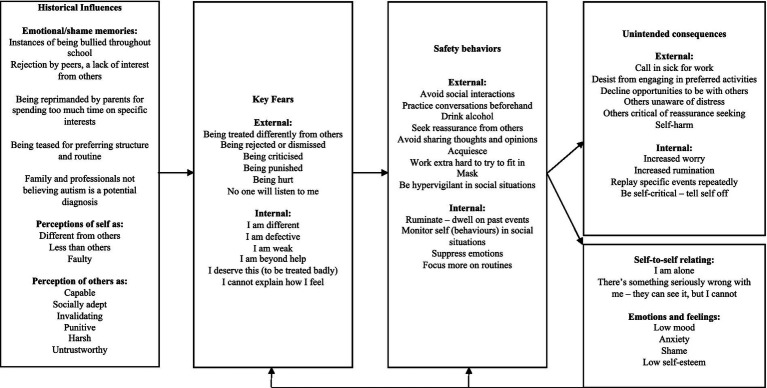
CFT formulation.

CFT helps to enhance qualities of compassion; *a caring commitment* to notice suffering, the *strength* to move toward it, and the *wisdom* to do what might help. This can help to cultivate a more self-caring, self-compassionate stance and a more fine-tuned emotion regulation system. Becoming more compassionate involves three processes: (1) kindness and curiosity, rather than a self-judging stance (*self-kindness* vs. *self-judgment*); (2) recognition we are all part of humanity, rather than alone (*common humanity* vs. *isolation*); and (3) having balanced thoughts rather than over-identifying with negative thoughts (*mindfulness* vs. *over-identification*) (Neff, 2003). Therapeutically, CFT is informed by collaborative development of an individualized threat-focused bidirectional formulation (Gilbert, 2022b; see Figure 1 for an illustrative example), in a space that feels safe, supportive and contained.

CFT encourages attention to three flows of a compassionate attitude: from the person to others (helping others), others to self (imagining a compassionate other who can provide this), and self to self (moving toward our own suffering) (Gilbert, 2022b). This is because many people thoughtfully and kindly extend compassion to others, but find accepting this or being compassionate toward themselves more tricky (Kirby et al., 2019). People are supported to enhance their capacity for (self)compassion, with interventions/techniques including experiential and imagery-based strategies, some of which are adopted from other approaches (e.g., cognitive behavior therapy, mindfulness) (see Table 1 for an overview).

**Table 1:** Compassion focused therapy with autistic adults: An overview of therapy phases, interventions and techniques.

Phases of CFT	Purpose of intervention and examples of techniques	Autism-informed adaptations
Psychoeducation: the flow of life	**Why**: introduce CFT model; outline evolutionary explanation for, and de-shaming of, present difficulties (e.g., ‘not your fault’, we did not choose to be born here, now, in this context)	Clarify whether shame is associated with thoughts about social interaction Identify strengths
Psychoeducation: 3 systems model	**Why**: introduce CFT model, explore the three emotion systems and notion that we have ‘tricky brains’; this can enhance flow of compassion to others as it raises the possibility that we all have different models of ourselves	Reduce complexity of language Use different colours or circle sizes to represent three systems
Developing self-soothing strategies	**Why**: learn how to activate and regulate the soothe system **How**: practice deep rhythm breathing; relaxation strategies; imagery exercises (e.g., safe/calm place, compassionate colouring); listen to relaxing music; participate in calming activities	Embed reasonable adjustments in self-soothing activities (e.g., to the temperature, sound, light) List enjoyable activities people can do alone or with others
Enhancing emotion recognition skills	**Why**: enhance recognition; highlight bidirectional relationships between emotions, behaviours and responses, in the self and others **How**: psychoeducation; behavioural experiments or tasks testing out experiences of, and reactions to, positive emotions; explore evolutionary explanations for each emotion and alternative behaviours	Enhance emotional literacy Use mindfulness to notice urges of different emotions Develop idiosyncratic scales to depict gradations of emotions (and associated physiological arousal) Draw pictures of different emotions to help externalise these
Addressing fear of compassion	**Why**: address compassion-interfering thoughts/beliefs (e.g., being compassionate is a sign of weakness), and primary emotions activated by compassion responses (e.g., worry/fear activated by kindness) **How**: Socratic conversations about past experiences and the meaning of these; cognitive interventions to target less helpful assumptions and beliefs	Provide psychoeducation about primary and secondary emotions Enquire about thoughts of meeting others; explore self-criticism as a potential attempt to change themselves or others; validate the emotions that arise from experiencing this Allow more time for behavioural experimentation Use visual/written summaries
Compassionate functional analysis	**Why**: identify links between: (1) influential experiences and events predisposing to shame-based difficulties; (2) key fears; (3) safety behaviours; and (4) intended and unintended consequences **How**: functional analysis	Provide examples if people struggle to identify consequences Repeat for different situations
Learning and practising: caring commitment	**Why**: to experience compassion in three behavioural attributes; to begin noticing suffering in oneself and others **How**: compassionate memory techniques; develop a ‘care box’, comprising objects that evoke calm or calm/relaxed memories	Use sensorily-adapted mindfulness to notice and slow down responses of getting stuck in, or avoiding, distress Socratic exploration of how others know we are suffering Use time-limited special interests to feel safe Include sensorily pleasurable objects in a ‘care box’
Learning and practising: strength	**Why**: to turn towards suffering and understand unintended consequences of turning away from suffering **How**: soothing rhythm breathing and safe place imagery to increase tolerance of powerful emotions; mountain meditation exercise	Incorporate new, helpful reflections into imagery or role-play to show strength Compare different postures in detail, using the mountain meditation exercise With consent, ask family to note changes in posture between sessions
Learning and practising: wisdom	**Why**: to have choice over how to live, approach suffering and alleviate this **How**: inner best friend exercise (treating/talking to yourself as you would a best friend); generate positive self-statements	Summarise positive statements on cue cards Use socratic dialogue about how to connect to others via less expressed emotions (i.e. when suffering)
Putting it all together	**Why**: develop an image of the self that is ‘equal, included and different’ **How**: consolidation of interventions above	Explore each of the three qualities above, from the perspectives of (i) receiving these, (ii) giving these to others and (iii) observing these between others Compile a flashcard of warning signs of becoming socially exhausted, and ways of getting adjustments
Developing social confidence	**Why**: enhance knowledge, skills and confidence in developing adaptive, secure affiliative relationships; consider whether self-criticism or shame impact upon interactions/relationships **How**: psychoeducation; chair work to explore different roles within different relationships; systemically-informed conversations about important past and current relationships	Clarify understanding of social relationships Consider drawing social circles with the person in the middle, family in a circle around them, and moving in circles further out, to identify friends, colleagues, acquaintances and strangers; make this visual – different coloured circles representing different types of relationship and responses to them Use systemic techniques to explore personal narratives and meaning-making about autism
Enhancing assertiveness	**Why**: to manage the wider context of adjustments needed for people to live well in work, relationships, health appointments and other situations **How**: chair work; role play; imagery exercises	Identify scenarios involving disclosing the autism diagnosis, to whom, and what this felt like Discuss thoughts/beliefs and emotions about masking – and the degree to which this may link to the formulation
Managing therapy endings	**Why**: for people to be empowered to make choices over how they live **How**: make a therapy blueprint; problem-solve potential setbacks; compassionate letter writing	Plan for gradual tapering of sessions Compile diagrammatic therapy blueprint or video/audio recordings of key points (use the therapist’s voice if this helps)

Reproduced by permission of Oxford University Press (Compassion Focused Therapy by Acland and Spain, 2022, p180–182, in Psychological therapies for adults with autism, edited by Spain et al., 2022). (Originally adapted from Gilbert, 2009a, b; Cowles et al., 2020).

## Risk factors for self-criticism and shame-based difficulties in autistic adults

Some autistic adults experience shame-based difficulties (e.g., Davidson et al., 2017, 2018; Gaziel-Guttman et al., 2023). Identifying risk factors for these is important for highlighting the need for systemic changes beyond the person and informing intervention approaches. We think there are several risk factors. However, we do not suggest that these outlined below comprise an exhaustive list. In a CFT context, we would advocate for ensuring autistic adults have a supportive and contained therapeutic space to talk about factors that may be pertinent for them, in order to help with processing difficult experiences and to inform choices about interventions.

Research evidence indicates that diagnostic clarity that a person is autistic is validating, contributes to self-acceptance (Hickey et al., 2018; Lilley et al., 2022), and enhances positive individual and community identity (Lilley et al., 2022). Challenges with autism assessment pathways (e.g., difficulty attaining a referral because professionals in mainstream services doubt an autism diagnosis, long waiting times) (Rogers et al., 2016), can prolong an inner struggle with sense of self, and lack of understanding about why a person engages as they do and has unique preferences that others do not share. This can contribute to feeling separate from, rather than together with, peers and a community. This can also contribute to feelings of shame.

Additionally, some autistic people mask or camouflage their interests, opinions or behaviors (Cook et al., 2021). Masking is described as aversive and effortful, and is associated with depression and feeling “inauthentic” (Bernardin et al., 2021; Hull et al., 2021). This, in turn, can lead to a paradoxical situation whereby the person strives to appear “normal,” and is, therefore, not believed to have difficulties – despite the toll masking takes. This can culminate in a disconnect between the internal and external worlds, exacerbating a sense of difference and a feeling of needing to hide aspects of the self.

Further autistic people are frequently victimized (Cooke et al., 2022; Trundle et al., 2022), directly or implicitly (Lim et al., 2022). Victimization negatively impacts psychosocial outcomes (Arseneault, 2018; Schoeler et al., 2018; Halliday et al., 2021) and increases vulnerability for shame-based difficulties. As in neurotypical samples (Matuschka et al., 2022), this may deter autistic people from seeking others out (to obtain help or for social reasons), and thereby, contribute to isolation and exacerbate marginalization. Also, some autistic people are socially naïve or vulnerable (Sreckovic et al., 2014), and may be taken advantage of or abused. Realizing this has happened is hugely upsetting, and can contribute to self-condemnation.

Invalidating environments and any resulting shame can negatively impact on attainment. Autistic people, for example, are less likely to succeed in education and employment despite high abilities and strengths (Chen et al., 2015; Black et al., 2022). Also, drop out from university is common (Cage and Howes, 2020). Repeated setbacks in these areas – which are deemed desirable and associated with status – can reinforce negative beliefs about being inept or a failure, give rise to a sense of humiliation and exacerbate a disconnect from others. This can also impact on confidence to seek out further employment opportunities.

Importantly, there are also potential systemic issues that can increase shame-based difficulties in autistic adults. For example, there is debate about how to conceptualize autism (e.g., as a disorder, condition, difference), and what language to use (e.g., person- or identify-first, ‘special interest’ or ‘passion’) (Bottema-Beutel et al., 2021; Monk et al., 2022). Reference to ‘disorder’ is medicalised, feasibly compounding worries/beliefs about being different.

Many autistic people experience stigma, including stereotypical views of autism and neurotypicality (Botha et al., 2022; Han et al., 2022). They may be pushed towards maths and science, for example, and deterred from doing arts, due to stereotypes about autistic peoples’ skills/abilities. Also, societally, there are stereotyped views about what comprises ‘normal’ behavior. Stimming (repetitive movements/actions), for instance, is an autistic trait. However, some autistic people are ‘told off’ or judged negatively for doing this, as though they are doing something inappropriate (Kapp et al., 2019). Similarly, talking about passions can result in negative or punitive responses (Stockwell et al., 2021). The impression that innate autistic traits are out of sync with societal norms, or that being autistic results in stigma, can exacerbate marginalization and disconnection.

Taken together, a range of individual and systemic risk factors potentially increase the development of CFT relevant shame-based difficulties and negative beliefs in autistic people, that can have real and distressing consequences on their lives and opportunities. Additionally, chronic invalidation can reduce the likelihood that autistic people will seek others out to ask for help or share their thoughts/worries and feelings. This indirectly, reduces options for gaining support and developing further strategies for processing the impact of difficult or upsetting experiences.

## Risk factors for a disrupted emotion regulation system

There are also possible vulnerability factors contributing to disruption in the CFT-relevant threat, drive and soothe systems for autistic people.

Neurobiologically, some research indicates that, relative to non-autistic people, autistic people may have features associated with autonomic dysfunction, such as decreased heart rate variability (Cheng et al., 2020), and differences in amygdala development and reactivity (Andrews et al., 2022). These potentially impact emotion regulation and contribute to a heightened threat system. More generally, autistic people are found to have higher rates of emotion dysregulation compared to neurotypical samples (see Cai et al., 2018 for review). This may also contribute to disruption in, for instance, threat and soothe systems.

Focusing on more psychologically and CFT-informed explanations, it may be that some autistic people develop a sensitized threat system early on in the context of social interaction (e.g., based on prior experiences of victimization, being ‘told off’ for getting things ‘wrong’), due to sensory overwhelm (e.g., to sound, light, and in response to anticipated and novel situations), or when needing to deal with uncertainty and change/transitions.

Alongside this, some autistic adults may have an over-active drive system. Difficult social experiences historically may result in attempts to ‘try harder’ (strive) to fit in (Leedham et al., 2020). This could involve masking thoughts and feelings or being in social situations they do not wish to be in. These are understandable reactions, but in the longer-term, this may reinforce a sense of threat, anxiety or worry. The drive system may be especially switched on for autistic people with no formal autism diagnosis, as unique preferences/needs may be invalidated by others. Additionally, there may be an interaction between the threat and drive system; some autistic adults may be driven towards doing activities and goals that are personally rewarding (e.g., passions, interests), but criticism from others about these amplifies a threat response, and thereby, a drive to compensate elsewhere.

Conversely, the soothe system may be disrupted or under-developed, but not out of choice. This may be due to the impact of traumatic events (Kerns et al., 2022), or lack of opportunity to develop reciprocal relationships. It may be that other people seem/are unsafe, or they are not nurturing in ways that fit the person’s preferences. Alternatively, being discouraged from stimming or following preferred routines may limit how much self-regulatory behaviors some autistic people engage in, and consequently, available self-soothing strategies. This could potentially give rise to a more threat-based, rather than soothe-based response. For example, stimming may indirectly switch on the threat system if others are negative/punitive about this. Another plausible consideration is the development of a neurodivergent soothe system. Autistic people may self-soothe in neurodivergent or different ways, such as through interests (and plausibly, routine) (Dachez and Nbodo, 2018). Disruption of these soothing strategies may increase threat system sensitisation.

## Autism, compassion, and CFT

To our knowledge, no studies have investigated CFT theory or interventions with autistic adults.

Four cross-sectional studies - with samples of up to 164 adults (Galvin et al., 2021; Howes et al., 2021; Cai et al., 2022; Galvin and Richards, 2022), and using self-report measures (including the Self-Compassion Scale; Neff, 2003) – and one qualitative interview study (*n* = 11; Wilson et al., 2022), have examined levels of self-compassion in adults with a formal autism diagnosis, self-identified autism and/or non-autistic adults, and associations between self-compassion, autistic traits and mental health. Together, quantitative findings suggest that autistic people, and people with autistic traits, report lower self-compassion than comparison non-autistic samples. Factors found to be associated with lower self-compassion in autistic people include age, education and gender; at group-level, these were not related to self-compassion in non-autistic people. Qualitative findings indicate that personal factors, notably having (waiting for) an autism diagnosis, masking and disclosure, could all worsen self-compassion.

## Systemic, clinical, and research implications

There are several systemic, clinical and research implications. Concerted societal efforts are needed to reduce stigma and discrimination (Botha et al., 2022; Han et al., 2022), and (in)direct victimization (Cooke et al., 2022; Lim et al., 2022; Trundle et al., 2022) towards autistic people. This would limit trauma exposure, and its impact. This may involve a move towards neurodiversity and strength-based paradigms. Additionally, enhancing the general understanding of autism by professionals seems key (Wood, 2020; Corden et al., 2022) for challenging inaccurate assumptions about autism/autistic people, and promoting concepts of difference/ability, rather than disability.

Improving access to, and the quality of, autism assessment pathways, and developing more accessible and acceptable autism-tailored health services is crucial; for example, finding out *from each person* if they would like adjustments/adaptations to an appointment context (e.g., reduce sensory stimuli, tailor communication style), the environment (e.g., an alternative to a sensorily overwhelming waiting room), and service provided (e.g., how appointments are arranged, language used in correspondence/patient records). Information about preferences and needs pertaining to health service use can be sought at different time points (e.g., in advance of the first appointment, but also at follow up). It is key that this is not a shaming experience; that is, the aim should be to find out how provision can be best tailored for each individual proactively, rather than making assumptions about what people will benefit from. This could reduce barriers autistic people encounter in health services (Nicolaidis et al., 2015), especially in mental health services (Brede et al., 2022), and overall health inequalities. It could also mean autistic people feel less pressure to mask or camouflage and are less in threat mode.

CFT can be offered as a standalone set of interventions, or as part of a phased therapeutic approach. Examples of autism-informed adaptations to CFT interventions are outlined in Table 1, and Acland and Spain (2022). Overall, the assessment phase of therapy should establish what helps to lessen activation of threat and drive systems and brings the soothe system online (e.g., encouraging stimming/self-regulatory activities, setting up the social context as the person prefers, making time to talk about passions and interests). Development of a formulation may take more time than service specifications usually afford for this. Also, negative beliefs, past adverse social experiences, a sense of shame and feeling the need to mask thoughts, can understandably impact the ease with which a person discusses aspects integral to formulation development. Therefore, psychological therapists should not rush this. Without taking the time needed in early phases of therapy, later phases are less person-specific, appropriately adjusted, accessible, and thereby, effective. Integral to the CFT approach is emphasis on the person’s strengths.

In terms of research implications, further studies should explore autistic people’s perspectives about the three flows of compassion and possible fears/blocks to any of these (e.g., being able to extend compassion, but difficulties tolerating this from others/themselves). Research focusing on mediating and moderating factors for low or high compassion would be informative (e.g., examining what constitutes a soothing relationship). The impact of shame-based difficulties is under-explored in autistic people, but seems a priority as poorer mental health contributes to difficulties across life domains. Finally, studies examining feasibility, acceptability and effectiveness of CFT interventions for autistic people, both proactively to enhance self-compassion and resilience, and reactively to target shame-based difficulties, are warranted.

## Conclusion

Difficult experiences, occurring through no fault of one’s own, can adversely impact mental health, give credence to negative beliefs and contribute to shame-based difficulties in some autistic adults. CFT can help to both make sense of underlying mechanisms for these, and provide a validating strengths-based framework for enhancing a person’s confidence and skills to feel able to be equal, included and themselves. Further research should examine the relevance of CFT principles and approaches with autistic adults. Moreover, we suggest there is an impetus for practitioners to consider how to optimally support autistic adults via a non-blaming and non-shaming approach.

## Data availability statement

The original contributions presented in the study are included in the article, further inquiries can be directed to the corresponding author.

## Author contributions

DM: Conceptualization, Writing – original draft, Writing – review & editing. JA: Conceptualization, Supervision, Writing – review & editing. ES: Conceptualization, Writing – review & editing. FH: Supervision, Writing – review & editing. DS: Conceptualization, Supervision, Writing – original draft, Writing – review & editing.
